# Citric acid-modified pH-sensitive bone-targeted delivery of estrogen for the treatment of postmenopausal osteoporosis

**DOI:** 10.1016/j.mtbio.2023.100747

**Published:** 2023-07-26

**Authors:** Zhong-Hua Chen, De-Yan Du, Yi-Fei Fu, Jun-Jie Wu, Dan-Yang Guo, Yue-Yue Li, Meng-Nan Chen, Zheng-Dong Yuan, Kai-Wen Zhang, Zhen-Yu Zhang, Xia Li, Feng-Lai Yuan

**Affiliations:** a Affiliated Hospital 3 of Nantong University, Medical School of Nantong University, Jiangsu, China; b School of Chemical and Material Engineering, Jiangnan University, Jiangsu, China; c Institute of Integrated Chinese and Western Medicine, Affiliated Hospital of Jiangnan University, Jiangsu, China

**Keywords:** Postmenopausal osteoporosis, Citric acid, Bone-targeted, pH-sensitive, Side effects

## Abstract

Bone targeted delivery of estrogen offers great promise for the clinical application of estrogen in the treatment of postmenopausal osteoporosis (PMOP). However, the current bone-targeted drug delivery system still has several issues that need to be solved, such as the side effects of bone-targeted modifier molecules and the failure of the delivery system to release rapidly in the bone tissue. It is important to aggressively search for new bone-targeted modifier molecules and bone microenvironment-responsive delivery vehicles. Inspired by the distribution of citric acid (CA) mainly in bone tissue and the acidic bone resorption microenvironment, we constructed a CA-modified diblock copolymer poly(2-ethyl-2-oxazoline)–poly(ε-caprolactone) (CA-PEOz) drug delivery system. In our study, we found that the CA modification significantly increased the bone targeting of this drug delivery system, and the delivery system was able to achieve rapid drug release under bone acidic conditions. The delivery system significantly reduced bone loss in postmenopausal osteoporotic mice with a significant reduction in estrogenic side effects on the uterus. In summary, our study shows that CA can act as an effective bone targeting modifier molecule and provides a new option for bone targeting modifications. Our study also provides a new approach for bone-targeted delivery of estrogen for the treatment of PMOP.

## Graphical abstract

**Figure:**
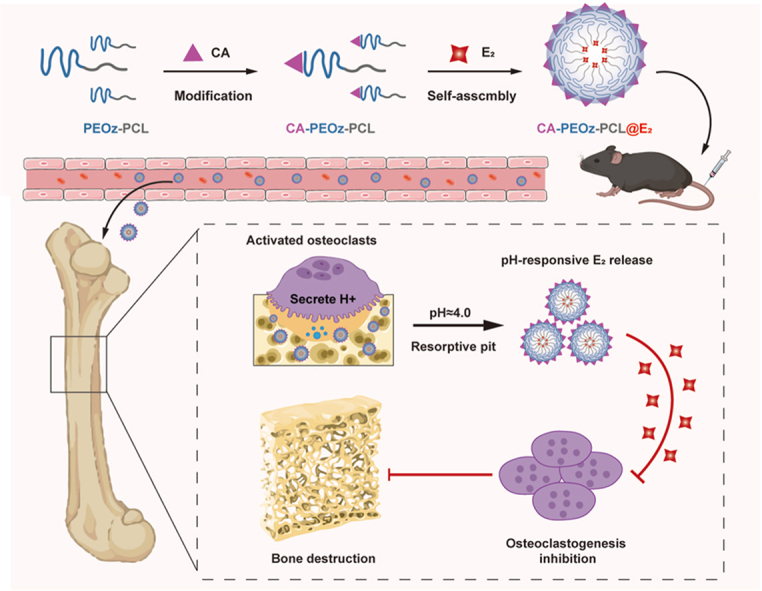

## 1 Introduction

Osteoporosis (OP) refers to a systemic skeletal disease related to bone-mass reduction and serious microarchitectural deterioration of the bone tissues [1]. Its prevalence in postmenopausal women is as high as 50% [2]. The leading reason for PMOP is postmenopausal estrogen deficiency, which disrupts the balance between bone formation and bone resorption by favoring the latter [3].

The current primary treatment for PMOP is drug therapy. Many therapeutic drugs, including calcitonin, bis-phosphonates, and receptor activator of nuclear factor kappa-β (RANK) ligand inhibitors, are effective in OP treatment [4]. Nevertheless, the abovementioned strategies generally focus on preventing bone loss and not targeting the initial cause of PMOP. A common estrogen drug, 17β-estradiol (E2), can address the initial cause of PMOP and decrease the incidence of osteoporotic fractures by around 50% in postmenopausal women [5,6]. Nevertheless, the traditional administration approaches of E2 can cause severe side effects, including high cancer risk in the breast, ovaries, and endometria [7,8]. To ameliorate the existing challenge, various types of nanomaterials were studied as ideal drug carriers, with polymeric micelles being the most widely studied and used ones [9,10]. Polymeric micelles have shown significant potential in increasing the water solubility of the drugs and prolonging the blood circulation time of the drugs [11]. To increase the polymeric micelle's affinity for bone tissue, their surfaces are introduced with various bone-targeting moieties, including bisphosphonates (BPs) [12], tetracyclines (TCs) [13], and peptides [14,15]. Nonetheless, as these bone-targeting moieties are not native to the body, they have many side effects. For example, BPs may result in osteonecrosis of the jaw and induce osteomalacia [16,17] and TCs leads to stained teeth and induces enamel hypoplasia during calcification [18,19].

Therefore, it is important to find a natural molecule with low immunogenicity and easy bioconjugation and synthesis, to modify the polymeric micelles and improve bone targeting. CA is a vital intermediate product of the tricarboxylic acid cycle in cell metabolism, and the majority of citrate (over 90%) is present in the bone tissues [20,21], which implies that CA possesses a high affinity for the bone tissues and the potential to mediate bone-targeted drug delivery. Additionally, the slow release of drugs from the polymeric micelles is another great concern because it can probably cause a lower level of free drugs in the bone tissues, thus, limiting the therapeutic efficacy. Thus, in order to ensure the delivery of the estrogen drugs to the bone tissues and sufficient drug concentrations for optimal efficacy, polymeric micelles are required to possess high stability in blood circulation and effective drug release to the target bone environment. Bone homeostasis is regulated by osteoblast formation and osteoclast resorption [22]. Mature osteoclasts adhere to the bone surface and secrete numerous hydrogen ions (H^+^) in order to form the local acidic extracellular microenvironment (pH = 4) for bone resorption [[22], [23], [24], [25]]. This acidic environment of the bone tissues can serve as a trigger for the rapid drug release from the polymeric micelles. Therefore, pH-sensitive polymeric micelles appear to be the most promising drug carriers.

Based on the aforementioned theoretical analysis, we successfully constructed a CA-modified and pH-sensitive polymeric micelle for estrogen therapy for PMOP. To the best of our knowledge, the use of the CA-modified poly(2-ethyl-2-oxazoline) (PEOz) has not yet been reported for bone-targeted drug delivery. This polymeric micelle showed a strong affinity for the bone tissues and achieved high bioavailability of E2, increasing the therapeutic efficacy as well as minimizing the negative impacts in the ovariectomy (OVX)-induced OP.

## 2 Materials and methods

### 2.1 Materials

HOOC-PEOz-PCL (Mw:5K), coumarin-6 (C6), indocyanine green (ICG), hydroxyapatite (HA) and tetraphenylethylene (TPE) were obtained from Guangzhou Carbon Water Technology Co., Ltd (Guangzhou, China). 4-dimethylaminopyridine (DMAP) and N,N′-Dicyclohexylcarbodiimide (DCC) were obtained from Aladdin Co., Ltd. (Shanghai, China). E2, tartrate-resistant acid phosphatase (TRAP) staining kit, and CA were bought from Sigma-Aldrich (St. Louis, MO). Macrophage colony-stimulating factor and receptor activator of NF-kappa B ligand were acquired from LifeTein (Beijing, China). Cell Counting Kit-8 (CCK-8), phosphate buffer saline (PBS) Masson stain kit, hematoxylin, and eosin staining kit, F-actin staining kit, Calcein AM/PI cell viability kit, and Hoechst 33,342 were acquired from Beyotime Biotechnology Co., Ltd. (Shanghai, China). Minimum essential medium α and fetal bovine serum (FBS) were provided by Gibco BRL (Carlsbad, CA). Other reagents of cell culture are purchased according to our previous research. C57BL/6 J mice were bought from Changzhou Cavens Laboratory Animal Co., Ltd. Bovine bone slices were obtained from JoyTech Bio Co., Ltd. (Hangzhou, China).

### 2.2 Synthesis and characterization of polymers

HOOC-PEOz-PCL (5 mg) was introduced into a round-bottom flask containing a mixture of dichloromethane, DAMP, and DCC. This mixture was stirred for 30 min at room temperature. Then, we introduced CA (0.5 mg) to elicit a reaction under ambient temperature for another 48-h period. Later, the sample was precipitated in cold diethyl ether and dried in a vacuum with the aim of obtaining the product.

Afterward, polymers acquired were subjected to dissolution in CDCl3; the Bruker MSL2300 spectrometer (400 MHz, Germany) was used to record ^1^H NMR spectra, with tetramethylsilane (TMS) being the endogenous control under ambient temperature for characterizing the product's chemical structure.

### 2.3 Preparation of blank HOOC-PEOz-PCL (PEOz) and CA-PEOz-PCL (CA-PEOz) polymeric micelles

CA-PEOz (5 mg) polymers were dissolved in chloroform (5 mL); the mixture was evaporated at 20 °C and in a vacuum to form a stripped film. Deionized water (10 mL) was introduced dropwise through constant stirring. Thereafter, the mixture was dialyzed (MWCO 3.5 kDa) for 4 h. Afterward, a 0.45-μm microporous membrane was used to filter the solution to obtain CA-PEOz micelles. PEOz micelles were similarly obtained.

### 2.4 Preparation of E2-loaded polymeric micelles

CA-PEOz (5 mg) polymers and 1 mg of E2 were dissolved in chloroform (5 mL) and evaporated at 20 °C and in a vacuum to form stripped films. Deionized water (10 mL) was introduced dropwise through constant stirring. Thereafter, the mixture was dialyzed (MWCO 3.5 kDa) for 4 h. Afterward, a 0.45-μm microporous membrane was used to filter the solution, followed by lyophilization to obtain CA-PEOz@E2 micelles. PEOz@E2 micelles were similarly obtained.

### 2.5 Drug encapsulation and release study

The obtained CA-PEOz@E2 micelles were dissolved in chloroform (5 mL), and the amount of β-estradiol loaded in the micelles was found by comparing the absorbance of the β-estradiol standard concentration curve at 280 nm in chloroform (Fig. S2). The encapsulation efficiency (EE) and drug loading efficiency (DLE) were identified based on the following equations, respectively:

$$EE\%=\frac{amount\;of\;E2\;in\;micelles}{total\;amount\;of\;E2}\times 100\%$$

$$DLE\%=\frac{amount\;of\;E2\;in\;micelles}{total\;weight\;of\;micelles}\times 100\%$$

In vitro drug release capability of CA-PEOz@E2 micelles was observed. CA-PEOz@E2 (5 mg) was resuspended in 1 mL PBS (pH = 4.0, 7.4). Subsequently, we transported this dispersion in the dialysis membrane bag [MWCO = 3500] that were dispersed into 10 mL PBS (pH = 4.0, 7.4) in the 37 °C water bath. At dedicated time points, we added 10 mL freshly prepared PBS to replace the original medium. High-performance liquid chromatography (HPLC) was carried out to measure the E2 release level.

### 2.6 Physicochemical characterization of polymeric micelles

The Malvern ZetaSizer Nano ZS (Malvern, UK) was used to detect polymeric micelles for their polydispersity index (PDI), hydrodynamic diameter, and zeta potential with dynamic light scattering (DLS) at the 90° scattering angle under temperature conditions of 25 °C after the micelle solution was diluted using deionized water to a suitable volume. In addition, a transmission electron microscope (TEM, JEM-1230, JEOL, Japan) was used to visualize micelle morphology. The zeta potential and size of CA-PEOz@E2 micelles were measured to evaluate the stability of micelles in FBS for 7 days.

### 2.7 Hemolysis assay

We harvested 1 mL of whole blood of mice into heparin sodium–containing tubes. Afterward, to remove plasma, blood samples were subjected to 10-min centrifugation at 1500 rpm and then rinsed thrice by prechilled PBS. In this step added 5 mL PBS for resuspending freshly collected red blood cells (RBCs) to prepare an RBC suspension. Further, 0.8 mL CA-PEOz solution of different concentrations was mixed with 200 μL RBC suspension. Sterile water (0.8 mL) was introduced into the RBC suspension (200 μL) to produce complete hemolysis as a positive control. PBS (0.8 mL) was then introduced into the RBC resuspension (200 μL) to create a negative control (NC). RBC suspensions were placed in the 37 °C water bath for a 12-h period, followed by 15-min centrifugation at 1000*g* to collect supernatants. At last, the absorbance (OD) value of hemoglobin released within 200 μL supernatants was measured at 570 nm. The following equation was employed to calculate the hemolysis ratio, with As, Anc, and Apc representing OD values of sample, NC, and positive control (PC), respectively:

$$Hemolysis\;ratio\left(\%\right)=\frac{As\;–\;Anc}{Apc-Anc}\times 100\%$$

### 2.8 Cytotoxicity study

The cytotoxicity of polymeric micelles was examined with the use of the pre-osteoblastic MC3T3-E1 cells and L929 cells. After polymeric micelles and the cells were cocultured for 1, 3, and 5 days, live−dead cell staining was conducted in line with the manufacturer's instructions. In the end, a confocal laser microscope (LSCM, LSM800, Zeiss, Germany) was used with the purpose of evaluating polymeric micelles.

CCK-8 was adopted for further identifying cytotoxicity. On days 1, 3, and 5, the CCK-8 reagent was supplemented into each well and incubated for 1 h based on the instructions of the manufacturer. Following the incubation, absorbance at 450 nm was measured via a Thermo Fisher Multiskan Mk3 microplate reader. Each measurement was repeated three times. In addition, the relative cell viability (%) was calculated by comparing it with the control wells that contained only the culture medium.

### 2.9 Cellular uptake assay

We cultivated MC3T3-E1 cells for a 24-h period within the culture dish. The medium that contained CA-PEOz-C6 and PEOz-C6 at the 200 ng mL^−1^ C6 concentration was used to replace the original medium. The medium was removed after incubating for 3 h, followed by PBS rinsing of cells thrice to eliminate unbound residue. Afterward, cell digestion and collection were completed using 0.25% trypsin. At last, 1 mL PBS was used to resuspend cells, and the C6 uptake was measured by FAScan flow cytometer. For qualitative assay using confocal laser scanning microscopy (CLSM), 4% PFA was added to fix cells following 4-h incubation. Thereafter, Hoechst 33,258 was used to stain cell nuclei, followed by imaging under CLSM.

### 2.10 In vitro osteoclast inhibition by CA-PEOz@E2

Fresh bone marrow macrophages (BMMs) were collected in 6- to 8-week-old C57BL/6 J mice according to a previous description and inoculated with 1 × 10^4^/well into a 96-well plate [26]. At 24 h, PBS that contained E2 (10^−7^ mol L^−1^), CA-PEOz, and CA-PEOz@E2 (10^−7^ mol L^−1^ E2) was supplemented to replace the medium. The medium was changed every two days, and TRAP staining (Wako-chemical, Japan) was performed after seven days of culture. Cells were rinsed in PBS thrice, followed by fixation for 30 min in 4% paraformaldehyde (PFA), in line with TRAP staining kit instructions. Images were taken by a microscope; the TRAP-positive osteoclast number was quantified.

### 2.11 Immunofluorescence imaging for visualizing podosomes belt

BMMs were cultured and later subject to fixation using 4% PFA and 15-min incubation with 0.1% Triton-PBS for membrane permeabilization. F-actin rings were stained with an F-actin staining kit for 30 min according to manufacturer instructions. After three washes, Hoechst 33,342 was used to stain DAPI for 10 min. A fluorescence microscope was used to observe and capture photos of DAPI and F-actin rings of osteoclasts.

### 2.12 Bone resorption assay

We cultured BMMs onto bovine bone sections within the 96-well plates. Following seven days of culture, a sonicator was used to remove the cells from the bovine bone slices, followed by natural air drying of plates for a 12-h period under ambient temperature. A scanning electron microscope (FEI Quanta 250, Hillsboro) was used to observe resorption pits onto bone sections, whereas ImageJ software was adopted for visualization. The pit area was calculated relative to the bovine bone section field area.

### 2.13 HA binding tests

PEOz and CA-PEOz micelles were labeled with TPE fluorescent probe first. Five mg CA-PEOz polymers and 1 mg TPE were dissolved in CHCl_3_ (5 mL), followed by evaporation under vacuum at 20 °C to form a stripped film. Deionized water (10 mL) was dropwise added under continuous stirring to form TPE-labeled CA-PEOz micelles. The mixed solution was dialyzed for 4 h. The whole process was conducted in the absence of light. TPE-labeled PEOz micelles were similarly obtained. Then, TPE-labeled PEOz and TPE-labeled CA-PEOz were diluted in 0.80 mg mL^−1^, and the initial fluorescence intensity was measured using fluorescence spectroscopy (λ_ex_ = 330 nm, λ_em_ = 460 nm, U = 700 V). Then, 25 mg HA was added to 4 mL TPE-labeled PEOz and TPE-labeled CA-PEOz. Binding experiments were conducted at 37 °C under continuous stirring (100 rpm) for 3 h. Subsequently, the suspension was centrifuged. The fluorescent intensity of the supernatant was also measured. TPE-labeled PEOz, as well as TPE-labeled CA-PEOz, amount bound to HA was calculated using the following equation:

$$HA\;absorption\;affinity\left(\%\right)=\frac{It0-It}{It0}\times 100\%$$

I_t0_ and I_t_ represent the initial fluorescent intensity and the fluorescent intensity measured from the supernatant, respectively.

### 2.14 Preparation of ICG-loaded and C6-loaded polymeric micelles

CA-PEOz-PCL polymers (5 mg) and ICG (1 mg) were dissolved in CHCl_3_ (5 mL), followed by evaporation under vacuum at 20 °C to form a stripped film. Deionized water (10 mL) was dropwise added under continuous stirring to form CA-PEOz@ICG micelles. The mixed solution was dialyzed for 4 h. The whole process was conducted in the absence of light. PEOz@ICG, CA-PEOz@C6, and PEOz@C6 micelles were similarly obtained.

### 2.15 In vivo biodistribution study

In order to assess the bone-targeting ability of CA-PEOz micelles, mice were administered with PEOz@ICG and CA-PEOz@ICG (ICG 2.0 mg kg^−1^) via tail intravenous injection. ICG biodistribution analysis was carried out with the 710-nm excitation wavelength and the 785-nm filter at 3 h and 6 h after injection, respectively. Moreover, the spine and hind limb were excised and detected in vitro for subsequent fluorescent quantitative analysis. Besides, femurs were collected and bone tissue sections were prepared to evaluate the distributions of PEOz@ICG and CA-PEOz@ICG in vivo.

### 2.16 Therapeutic assay of osteoporosis in vivo

We obtained 32 female C57BL/6 mice (7-week-old) from Changzhou Cavens Laboratory Animal Co., Ltd. Each experiment was carried out following the regulations for the administration of affairs concerning experimental animals. Ovariectomy was performed after the mice were completely anesthetized based on an intraperitoneal injection of 1% pentobarbital (0.1 mL/100 g). Thereafter, animals were randomized into four groups, with five each, including sham mice (ovary intact) that received PBS treatment (sham), OVX mice that received PBS treatment (OVX), OVX mice that received E2 (E2) treatment, and OVX mice that received CA-PEOz@E2. At 1 week of postoperative recovery. The injection was given in 8 weeks every second day through the tail vein. In the OVX + E2 and OVX + CA-PEOz@E2 groups, E2 doses were administered at 20 μg kg^−1^ body weight (BW). Our animal experimental protocols gained approval from the Laboratory Animal Committee of Jiangnan University.

### 2.17 Micro-computed tomography

In the μCT analysis, we dissected the right femoral samples and immersed them in 10% neutral-buffered formalin post-treatment. Later, we conducted 3D reconstruction based on 2D images by adopting a 3D creator software for the instrument. Within the volume of interest (VOI), the corresponding software was used to analyze 3D indexes, including bone volume over tissue volume (BV/TV), bone mineral density (BMD), trabecular separation (Tb.Sp), trabecular thickness (Tb·Th), trabecular number (Tb·N), cortical thickness (CT.Th) and cortical cross-sectional area (CT.Ar). Milligrams of HA per cubic centimeter (mg HA/ccm) was the unit of measurement.

### 2.18 Biomechanical parameter analysis

Following μCT analysis, the three-point bending test machine (Instron Corporation, Norwood, MA) was used to investigate right femoral biomechanical indexes. Two bottom supports were separated at a spacing of 10 mm. In each test, the metal pole was placed in the middle of two supports, whereas the metaphysis loading speed was set at 1 mm/min. According to the load–deformation curve, biomechanical indexes were recorded, including Young's modulus (MPa), maximum strength (MPa), and maximum load (N).

### 2.19 Histological staining of bone tissue

The tibias of mice were gathered and immersed in a 10% neutral formaldehyde solution for fixation for 24 h. Tibial samples were subject to 1-month decalcification with 10% EDTA–glycerol solution. The dehydrated tibias were later subjected to paraffin embedding, followed by slicing to 5-μm sections for H&E, Masson, and TRAP staining according to respective manufacturer instructions.

### 2.20 Detection of bone turnover markers in serum

In order to extract serum, the collected blood samples were centrifuged for 10 min. Bone turnover markers, *N*-terminal pro-peptide of type-I procollagen (PINP), and C-terminal telopeptide-I (CTX-I) in the serum were evaluated using enzyme-linked immunosorbent assay (ELISA) in line with manufacturer instructions (Shanghai Enzyme-linked Biotechnology Co., Ltd.).

### 2.21 Systemic toxicity of CA-PEOz@E2

To further investigate cytotoxicity in vivo, serum and whole blood were gathered for performing the analysis at the end of treatment. Using a hematology autoanalyzer, white blood cell (WBC), red blood cell (RBC), hemoglobin (HGB), as well as blood platelet (PLT) were measured. In addition, this study also identified serum alanine aminotransferase (ALT), aspartate aminotransferase (AST), creatine kinase (CK) and creatinine (Cr) contents. Moreover, the major organs (heart, liver, spleen, lung, and kidney) of mice were gathered and prepared for H&E staining to evaluate systemic toxicity.

### 2.22 Statistical analyses

All quantitative data are values of means ± standard deviation. We used Student's *t*-tests to perform comparisons. Mann–Whitney *t*-test was adopted for nonparametric analysis. Comparisons between three or more groups were carried out with one-way analysis of variance (ANOVA) or two-way ANOVA based on various comparison tests (Tukey's test). By adopting the GraphPad Prism software, statistical significance was decided. Values of *P < 0.05, **P < 0.01, and ***P < 0.001 were thought to be of statistical significance.

## 3 Results

### 3.1 Synthesis and characterization of CA-modified-PEOz–poly(ε-caprolactone) (CA-PEOz-PCL) conjugates

CA-PEOz-PCL conjugates were successfully synthesized through an amide reaction between the carboxyl group of HOOC-PEOz-PCL and CA. The fundamental chemical structure of the polymer was confirmed by proton nuclear magnetic resonance (^1^H NMR) (Supplementary: Fig. S1).

### 3.2 Preparation and characterization of E2-Loaded CA-PEOz-PCL micelles (CA-PEOz@E2)

Appropriate drug load has a vital role in the therapeutic effect of polymeric micelles. Increasing the E2 amount in CA-PEOz-PCL led to a higher loading efficiency of E2, whereas the drug loading efficiency (DLE) of the polymeric micelles decreased. To determine the optimal ratio of E2 and CA-PEOz-PCL, the ratio of E2 and CA-PEOz-PCL was increased from 1:15 to 4:10. The data showed that the encapsulation efficiency (EE) of E2 reduced from 93.34% to 48.79% and DLE increased from 5.91% to 14.37% (Table S1). When the ratio of E2 and CA-PEOz-PCL was increased from 1:15 to 2:10, DLE of E2 increased from 5.91% to 13.19%. However, a further increase in the ratio did not increase the DLE of E2, but EE markedly decreased. Therefore, 2:10 was determined as the optimal ratio of E2 and CA-PEOz-PCL for further researches. At this ratio, EE of CA-PEOz@E2 was 77.72% and DLE was 13.19%.

**Fig. 1:**
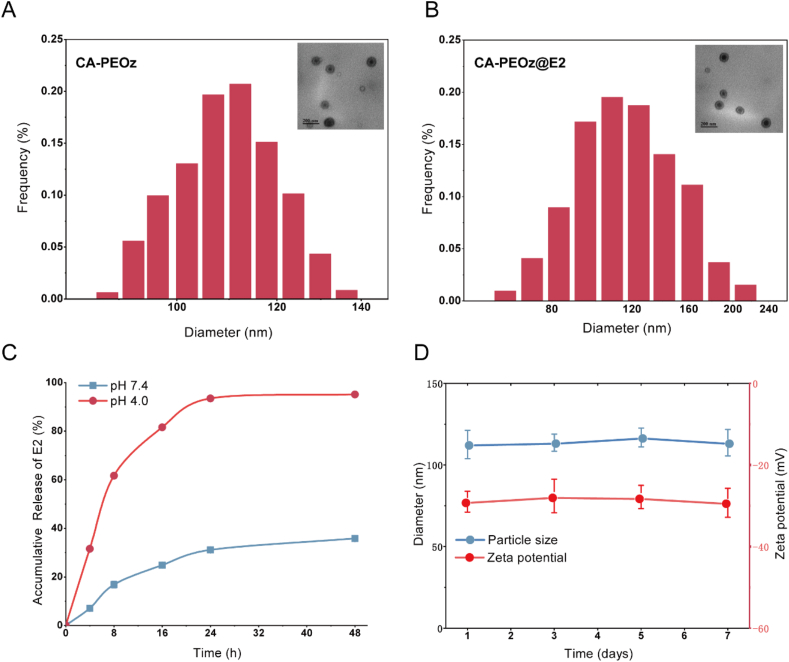
Characterization of CA-PEOz@E2 (A) Size distribution and morphological features of CA-PEOz. (B) Size distribution and morphological characteristics of CA-PEOz@E2. (C) In vitro E2 release profiles of CA-PEOz@E2 at different pH (7.4 and 4.0) (n = 3). (D) Stability evaluation of CA-PEOz@E2 during a week of storage by adopting DLS analysis (n = 3).

Dynamic light scattering (DLS) characterization showed the hydrodynamic diameter and zeta potential of CA-PEOz@E2 were around 119.5 nm and −27.4 eV, respectively. The physicochemical properties of the associated micelles were also characterized in this study (Table S2). Size distribution and morphological characteristics of CA-PEOz (Fig. 1A) and CA-PEOz@E2 (Fig. 1B) showed that CA modification slightly increased the particle size of micelles. In addition, the in vitro release behavior of CA-PEOz@E2 micelles at 37 °C was investigated at pH 7.4 and pH 4.0. E2 was continuously released from CA-PEOz@E2 for 48 h, reaching the release rate of 95.1% at pH 4.0 (Fig. 1C). Comparatively, the release rate of E2 at pH 7.4 merely reached 35.8%. These results showed that E2 was released from CA-PEOz@E2 in a pH-dependent manner. The pH-dependent drug release could prevent drug leakage in the bloodstream circulation and increase the drug release at the osteoclast microenvironment.

To determine the stability of CA-PEOz@E2 micelles, they were redispersed in PBS at room temperature. Using DLS, the particle size and the surface zeta potential of CA-PEOz@E2 were constantly detected for a week. No significant changes were found in the particle sizes and the surface zeta potential, indicating that CA-PEOz@E2 had considerably high stability (Fig. 1D).

### 3.3 In vitro biocompatibility of polymeric micelles

**Fig. 2:**
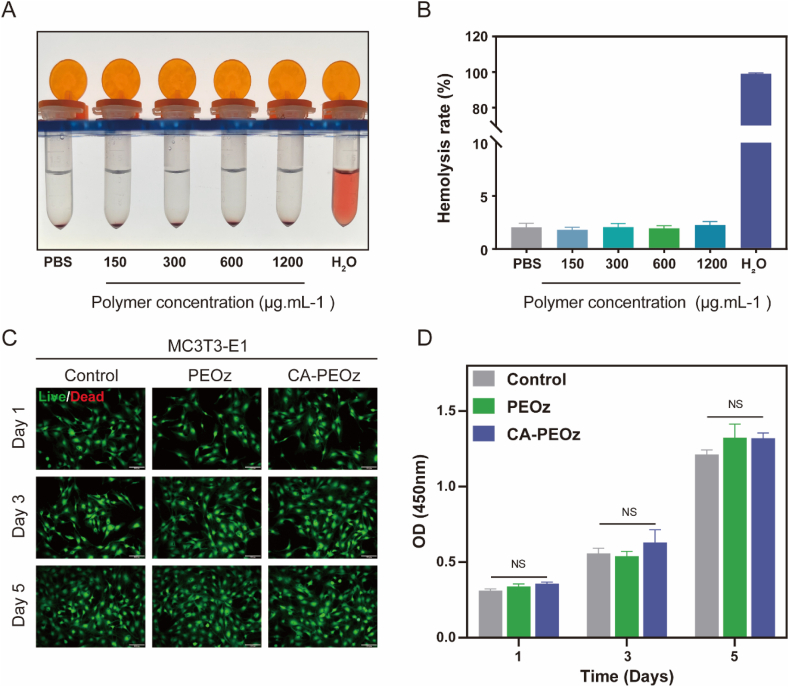
Biocompatibility of polymeric micelles (A) Co-incubation pictures of RBCs with different concentrations of CA-PEOz micelles. (B) The hemolysis ratio of RBCs was calculated at different concentrations of the CA-PEOz micelles (n = 3). (B) MC3T3-E1 cells cocultured with PEOz and CA-PEOz for 1, 3, and 5 days and explored by live/dead staining. Live cells are green; dead cells are red. Scale bars represent 100 μm. (C) Cell viability of MC3T3-E1 cells cocultured with PEOz and CA-PEOz for 1, 3, and 5 days by CCK-8 assay (n = 3). NS mean no significant.

Excellent biocompatibility is an essential requirement for the biomedical application of micelles. To investigate the biocompatibility of the CA-PEOz@E2 micelles, the hemolysis assay of the red blood cells (RBCs) was first performed to quantify the properties of the materials accounted for leading to membrane damage. The hemolysis rate of CA-PEOz was evaluated at varying concentrations within the range of 150–1200 μg/mL (Fig. 2A and B). None of the groups presented visible hemolytic activities within the tested concentration range. Furthermore, the live/dead staining and Cell Counting Kit-8 were adopted for determining the cytotoxicity of the polymeric micelles for 1, 3, and 5 d. The results of the live/dead staining showed that PEOz and CA-PEOz was less toxic when cocultured with MC3T3-E1 cells (Fig. 2C). Using CCK-8 among each group, the cell viability was further quantitatively assessed MC3T3-E1 (Fig. 2D) and L929 cells (Fig. S4). Quantitative statistical analysis indicated that CA-PEOz and PEOz have good biocompatibility. Collectively, the obtained findings suggested that the polymeric micelles exhibited good biocompatibility and low cytotoxicity to the normal cells.

### 3.4 Cellular uptake capability of the polymeric micelles

**Fig. 3:**
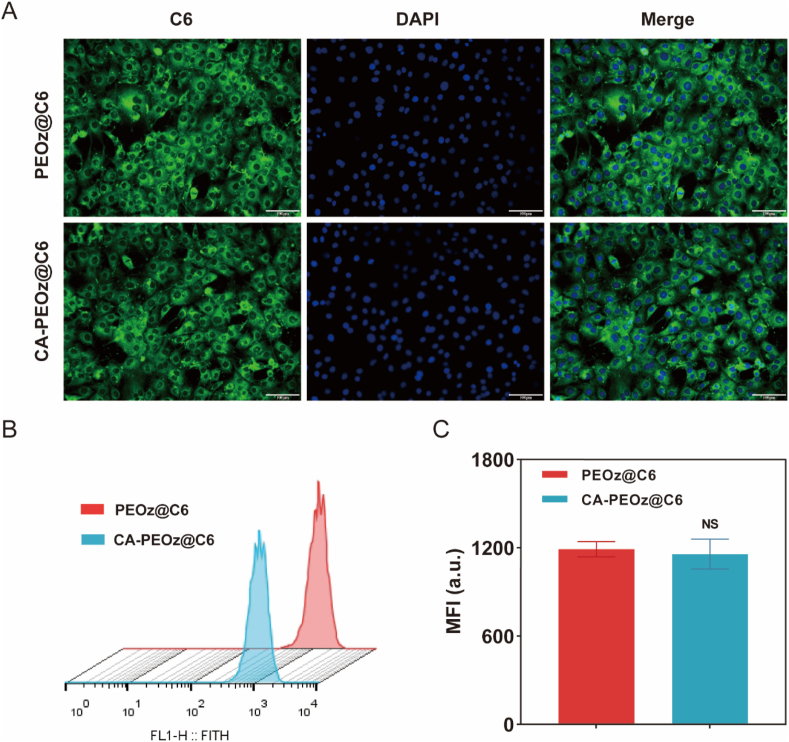
In vitro cell uptake capability of polymeric micelles. (A) Confocal microscopy images of MC3T3-E1 cells after 4 h incubation with PEOz@C6 and CA-PEOz@C6. Polymeric micelles were labeled by fluorescent dye C6. Cell nuclei were stained with Hoechst 33,342. (B, C) Quantitative flow cytometry analysis results of MC3T3-E1 cells cocultured with PEOz-C6 and CA-PEOz-C6 for 4 h (n = 3). NS mean no significant.

Coumarin-6 (C6, green) fluorescent label was used for tracking the cellular uptake. Firstly, the cellular uptake capabilities of numerous micelles were visualized by confocal laser scanning microscopy (CLSM). All drug formulations caused fairly strong green fluorescence near the cytoplasm and closely accumulated near the nuclei (blue) of MC3T3-E1 cells (Fig. 3A). According to the results, the uptake of the polymeric micelles was in good amounts. The cellular uptake was further quantified via flow cytometry (Fig. 3B and C). No significant difference existed in the fluorescence intensity of C6 for PEOz and CA-PEOz. This result indicated that CA modification on the micelle surface did not affect the cellular uptake of PEOz by MC3T3-E1 cells.

### 3.5 CA-PEOz PEOz@E2 inhibited osteoclast formation and bone resorption in vitro

**Fig. 4:**
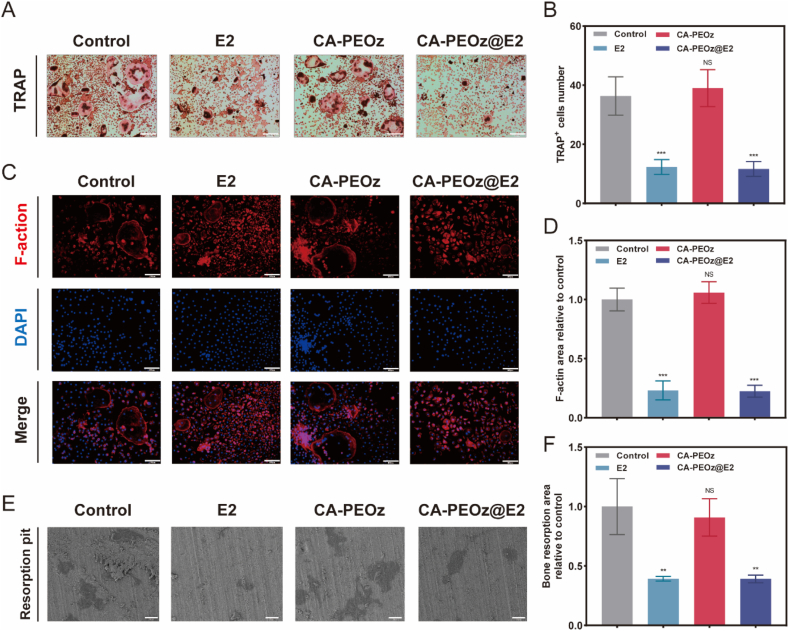
Effects of CA-PEOz@E2 on inhibition of osteoclasts in vitro. (A) BMMs were treated in different groups. TRAP staining was adopted for observing the formation of osteoclasts. Scale bars represent 200 μm. (B) The number of TRAP^+^ cells in different groups (n = 3). (C) The F-actin rings and cell nuclei were stained separately and observed using a confocal microscope in different groups. Scale bars represent 100 μm. (D) The area of F-actin rings in different groups is calculated in comparison with that in the control group (n = 3). (E) Using a scanning electron microscope (SEM), an image of bone resorption pits is shown. Scale bars represent 20 μm. (F) Areas of the resorption pit measured in different groups in comparison with those in the control group (n = 3). NS mean no significant. *: P < 0.05 and **: P < 0.01 and ***: P < 0.001.

Excessive increase in bone resorption by osteoclasts is an important characteristic of PMOP. Therefore, the effects of CA-PEOz@E2 on osteoclast formation and bone resorption capacity were investigated. TRAP staining confirmed that the CA-PEOz@E2 and E2 group greatly decreased the number of osteoclasts (Fig. 4A and B). F-actin rings, one of the components of the sealing zones, can affect the bone resorption capacity of osteoclasts. We observed that the CA-PEOz@E2 and E2 groups presented a smaller area of F-actin rings compared with the CA-PEOz and control group (Fig. 4C and D). To further investigate the osteoclastic bone resorption capacity on the bone surface, a bovine bone resorption assay was carried out. Significant reductions in the bone absorption pit areas were found in the CA-PEOz@E2 and E2 group via scanning electron microscopy (SEM) (Fig. 4E and F). To conclude, these results suggested that CA-PEOz@E2 inhibited the formation and bone resorption of osteoclasts.

### 3.6 Bone mineral binding ability and in vivo biodistribution study

**Fig. 5:**
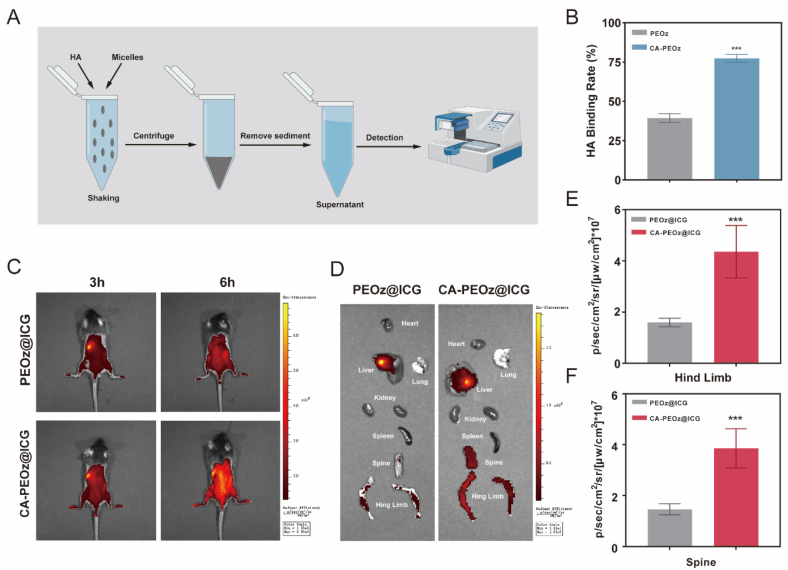
Bone-targeted ability of CA-PEOz micelles. (A) Schematic of HA adsorption affinity experiment. (B) In vitro HA binding capacities of CA-PEOz and PEOz micelles. (C) In vivo fluorescence images of mice after tail vein injection of CA-PEOz-ICG and PEOz-ICG at 3 h and 6 h, respectively. (D) Fluorescence images of ex vivo organs (including heart, liver, spleen, lung, kidney, spine, and hind limb) at 6-h post-injection. (E–F) Fluorescence intensity quantification of isolated hind limbs and spines (n = 5). NS mean no significant. *: P < 0.05, **: P < 0.01, and ***: P < 0.001.

High binding activity to the bone tissues remained a prerequisite for bone-targeted delivery. By comparing the HA binding capacity of PEOz and CA-PEOz, the in vitro bone affinity was assessed. The findings indicated that a higher proportion of CA-PEOz micelles bound to HA in comparison with the PEOz micelles (Fig. 5B), which demonstrated the bone targeting ability of CA-PEOz. To further validate the bone targeting efficacy of the CA-PEOz micelles in vivo, indocyanine green (ICG)-loaded polymeric micelles (PEOz@ICG and CA-PEOz@ICG) were intravenously injected into the mice and fluorescence images were taken. At 3 h after injection, we observed no significant differences in the distribution of the extremities (Fig. 5C). The fluorescence intensity of CA-PEOz@ICG quickly increased in the bone tissues (spine and hind limb) after 6 h. To further quantify the data, we isolated the spine and hind limb signal intensities at 6 h (Fig. 5D). The fluorescence intensity quantification showed that the spine and hind limb of the CA-PEOz@ICG group had a stronger fluorescent signal compared with that of the PEOz@ICG group (Fig. 5E and F). In terms of bone histological distribution (Fig. S5), CA-PEOz@ICG group had a stronger fluorescent signal compared with that of the PEOz@ICG group. According to the results, the CA modification could notably improve the bone-targeting ability of the polymer.

### 3.7 The therapeutic effect of the CA-PEOz@E2 micelles in the OVX mice

**Fig. 6:**
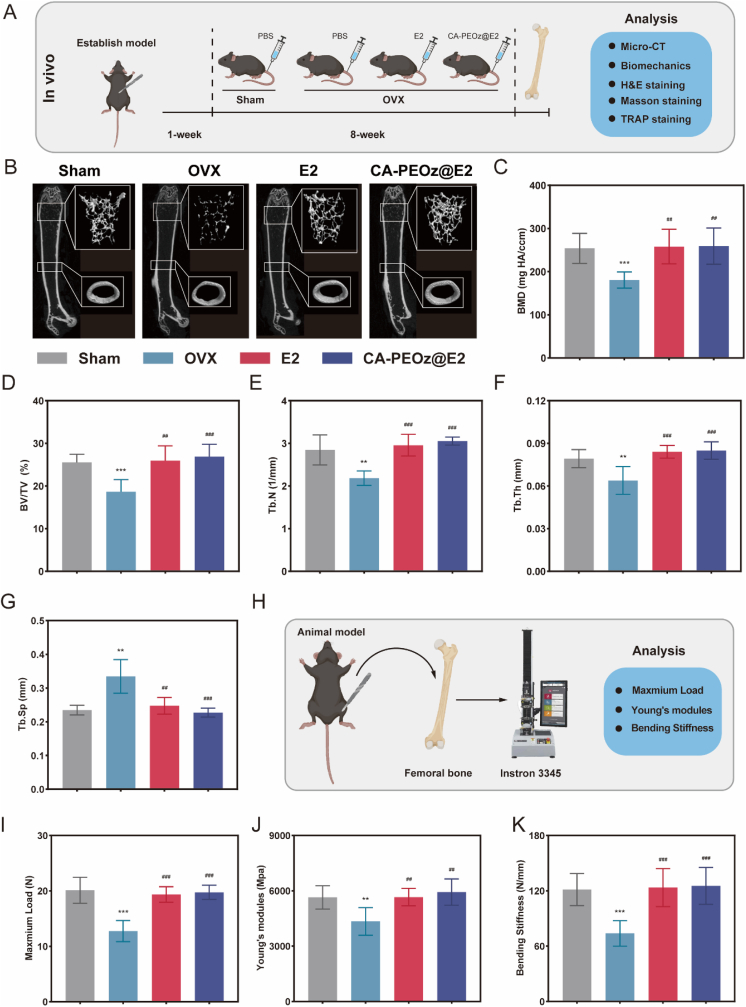
Protection of CA-PEOz@E2 from OVX-induced bone loss. (A) Schematic of the establishment of the OVX mouse model and the experimental design in order to assess the protective impacts of CA-PEOz@E2. (B) Micro-CT reconstruction images of the distal femurs of the experimental mice and the quantitative data of (C) BMD, (D) BV/TV, (E) Tb·N, (F) Tb·Th, and (G) Tb· Sp. (H) Schematic of femur biomechanical experiment. (I) Maximum load, (J) Bending stiffness, and (K) Young's modules detected in each group. n = 8. NS mean no significant. *: P < 0.05, **: P < 0.01, and ***: P < 0.001. vs. the Sham. #: P < 0.05, ##: P < 0.01, and ###: P < 0.001. vs. the OVX group.

To assess the therapeutic effects of the CA-PEOz@E2 micelles, OVX osteoporotic mouse model was used. Mice were classified into the following four groups: sham, OVX, E2, and CA-PEOz@E2 groups. Three-dimensional bone images and quantitative data on bone microstructure changes were analyzed by micro-computed tomography (Fig. 6B–G). The OVX group presented an obvious bone-mass loss compared with the sham group. The parameters of bone mineral density (BMD), bone volume over tissue volume (BV/TV), trabecular number (Tb·N), and trabecular thickness (Tb·Th) exhibited an obvious decrease, and trabecular separation (Tb.Sp) presented a significant elevation in the OVX group, suggesting the successful establishment of PMOP. In relative to the OVX group, the bone-mass increase was observed in the E2 and CA-PEOz@E2 treatment groups. BMD significantly recovered to around normal levels in the E2 and CA-PEOz@E2 treatment groups. The bone cortical data (CT.Th and CT. Ar) are consistent with the above results (Fig. S6). Excellent biomechanics is an essential requirement for ensuring the normal functioning of the bones. The biomechanical properties of the bone tissues were evaluated in each group. Based on the results, the maximum load, bending stiffness, and Young's modulus were significantly higher in the E2 and CA-PEOz@E2 groups compared with the OVX group (Fig. 6I–K).

**Fig. 7:**
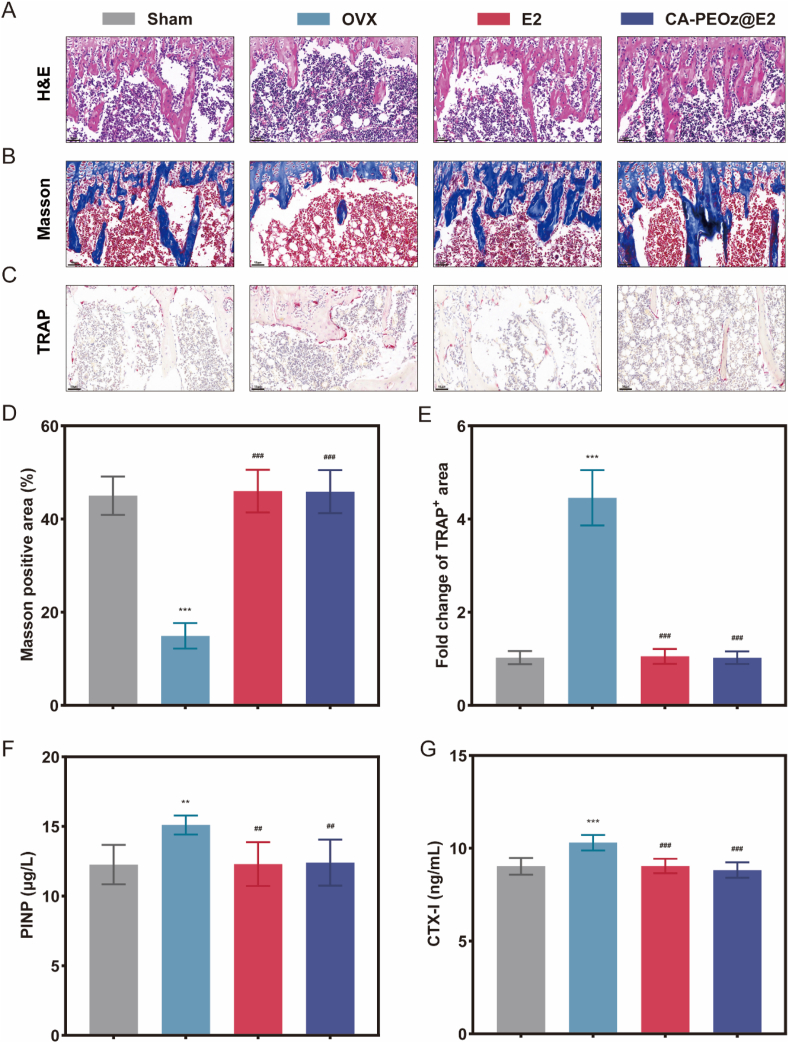
Histological evaluation of bone after CA-PEOz@E2 treatment. (A) H&E staining and (B) Masson staining of the left femur to observe bone microarchitecture. (C) TRAP staining of the left femur is performed to evaluate the effect of CA-PEOz@E2 on osteoclasts. Scale bars represent 50 μm in A, B and C. (D) Quantifications of Masson and (E) TRAP-positive area in each group. Effects of CA-PEO@E2 on bone turnover biomarkers in OVX mice, containing (F) serum PINP and (G) serum CTX-I. n = 8. NS mean no significant. *: P < 0.05, **: P < 0.01, and ***: P < 0.001. vs. the Sham. #: P < 0.05, ##: P < 0.01, and ###: P < 0.001. vs. the OVX group.

Additionally, histological analysis was also carried out to evaluate the impact of the CA-PEOz@E2 micelles on the bone. Consistently, hematoxylin and eosin (H&E) and Masson staining indicated that the trabeculae of the OVX group were significantly thinner and in a disordered arrangement (Fig. 7A, B and D). The pathological changes were alleviated in the E2 and CA-PEOz@E2 groups. TRAP staining showed that the number of osteoclasts (TRAP-positive cells) in the OVX group significantly elevated in comparison with the sham group (Fig. 7C and E). The osteoclastogenesis of the E2 and CA-PEOz@E2 groups was inhibited compared with that of the OVX group. The bone turnover markers, showing bone formation and resorption, were also tested to evaluate the efficacy of the CA-PEOz@E2 micelles (Fig. 7F and G). The serum PINP and CTX-Ι levels were elevated in the OVX group in relative to the sham group. Their levels exhibited a noticeable decrease in the OVX mice after treatment with E2 and the CA-PEOz@E2 micelles. Altogether, these data indicated that the CA-PEOz@E2 treatment effectively improved OVX-induced OP in the mice.

### 3.8 Side effects on the uterus

**Fig. 8:**
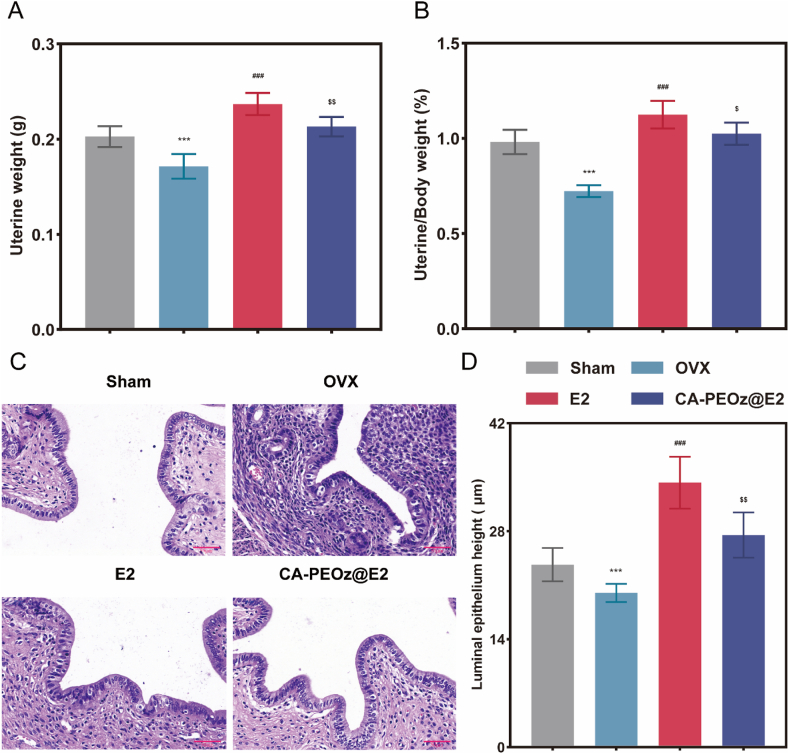
Effects of CA-PEOz@E2 treatment on the uterus in OVX mice. (A) Uterine wet weight and (B) the ratio of uteruses/body weight in different treatment groups. (C) Histological images of the endometrium in different treatment groups after H&E staining. Scale bars represent 50 μm. (D) Uterine luminal epithelial height in each group (n = 8). NS mean no significant. *: P < 0.05, **: P < 0.01, and ***: P < 0.001. vs. the Sham. #: P < 0.05, ##: P < 0.01, and ###: P < 0.001. vs. the OVX group. $: P < 0.05 and $$: P < 0.01. vs. the E2 group.

Because of nonspecific interactions, endometrial thickening and carcinoma are serious side effects of the E2 treatment. Therefore, an excellent target material can not only prevent osteoporosis but also reduce uterine side effects. Further, the effect of CA-PEOz@E2 micelles on the uterus was evaluated. The results showed that both uterine wet weight and the ratio of uteruses/body are statistically reduced in the OVX group compared with that in the other groups (Fig. 8A and B). In addition, the uterine wet weight and the ratio of uteruses/body are significantly higher in the free E2-treated group than in the CA-PEOz@E2 group. Furthermore, we used H&E staining to measure the uterine luminal epithelial height (Fig. 8C). Quantitative data showed that the luminal epithelial height was significantly increased in the E2 group relative to that in the sham and OVX groups (Fig. 8D). In addition, the luminal epithelial height was decreased in the OVX + PBS and CA-PEOz@E2 groups. CA-PEOz@E2 micelles, thus, show less association with uterine tissue and reduce the side impacts of E2.

### 3.9 Safety assessment of polymeric micelles in vivo

At the experimental endpoint, we performed body weight, hematological and histological analyses to assess the biocompatibility of CA-PEOz@E2 micelles in vivo. The body weight of CA-PEOz@E2 micelles have no significant difference compared to E2 group (Fig. S7). WBC, RBC, HGB, and PLT were in the normal value range and had no significant difference in all groups (Fig. S8). Serum biochemistry indexes, including ALT, AST, CK, and CR, were also detected, with no obvious significant changes in all groups (Fig. S9). In contrast, no significant pathological changes were found in the heart, liver, spleen, lung, and kidney in all groups (Fig. S10). Thus, CA-PEOz@E2 micelles possess biocompatibility in treating OVX mice.

## 4 Discussion

Osteoporosis, a common bone disease, features an imbalance in bone formation and bone resorption, generating the deterioration of bone microarchitecture, low bone density, as well as poor bone strength [27]. PMOP, a result of estrogen deficiency, is the most common category of osteoporosis [28,29]. Many drugs have been studied to reverse bone loss; however, little attention has been focused on solving the initial cause of PMOP [30]. E2 has been broadly used as an estrogen replacement therapy drug for the prevention and treatment of PMOP [31]. Nevertheless, due to poor bone targeting and severe side effects, clinical use of E2 has been critically restricted during the past decade. In this study, we constructed a CA-modified pH-sensitive bone-targeted delivery of estrogen for the treatment of PMOP. Unlike previously-reported bone-targeted delivery of estrogen for osteoporosis treatment, we chose a natural small molecule CA modification for this bone targeted delivery. Xue et al. constructed a BP-modified bone-targeted delivery miRNA system for the treatment of osteoporosis [32]. Que et al. reported TC-grafted for a bone-targeted drug-delivery system to treat osteoporosis [13]. BPs may result in osteonecrosis of the jaw and induce osteomalacia [16,17]and TCs leads to stained teeth and induces enamel hypoplasia during calcification [18,19].

CA is a vital intermediate product of the tricarboxylic acid cycle in cell metabolism. It strongly binds to Ca2+ and mineral surfaces of bone, a result of interaction between acidic carboxyl residues and these substances [33]. To the best of our knowledge, this is the first report of using CA as a bone targeting modifier molecule. Our results show that the CA-modified vector has good bone targeting properties. Many previous studies have used BPs and TCs as bone-targeting carriers for drug delivery, but many of them did not investigate the comparison of side effects on non-bone organs using the drugs alone. Our study also indicate that CA-modified carriers significantly reduce side effects of the uterus in E2 treatment of osteoporosis.

On the other hand, considering the acidic environment that develops when osteoclasts undergo bone destruction, we chose the pH-dependent micelles PEOz. In the absence of osteoclast adhesion, CA-modified PEOz can target the bone surface and the drug is released slowly to act. As osteoclasts adhere to the bone surface to form an acidic bone resorption microenvironment, this promotes rapid drug release in the bone tissue. Although we were unable to demonstrate this process in vivo, Lin et al. used TC-modified bone-targeted delivery to the bone surface to neutralize the acidic environment during osteoclast bone resorption for the purpose of improving bone mass [34]. These demonstrate that drug delivery to normal bone surfaces can take advantage of the acidic microenvironment of bone resorption to release the drug.

Of course, we have to admit that the experiments have some limitations. Although our experiments comply with the 3 R principles of animal ethics, our limited number of animal experiments may affect the credibility, but our experimental results are sufficient to support our current conclusions.

## 5 Conclusions

We successfully constructed a bone-targeting and pH-responsive nanocomposite (CA-PEOz@E2) that served as a drug-delivery system. These micelles exhibited strong stability, good biocompatibility, and bone-targeted ability. In addition, we explored their impact on suppressing osteoclast formation in vitro. In vivo, these micelles prevented osteoporosis induced by ovariectomy and had fewer side effects on the uterus than E2. The appeal of this system is that the novel CA-modified and pH-responsive drug-delivery strategy not only expands the concept of bone-targeting modification but also promotes drug release in the bone resorption microenvironment. Moreover, this study also offers a promising strategy for bone targeting modification and provides a reference for hormone drug delivery in the future.

## Credit author statement

Z-H Chen and F-L Yuan generated themes and ideas. Z-H Chen, D-Y Du, Y-F Fu and D-Y Guo conducted experiments and drafted the manuscript. J-J Wu, Y-Y Li, M-N Chen, Z-D Yuan and K-W Zhang prepared the figures. Xia Li and F-L Yuan discussed and revised the manuscript.

## Declaration of competing interest

No conflicts of interest have been declared by any authors.

## Data Availability

Data will be made available on request.
