# Supplementary material for: Citric acid-modified pH-sensitive bone-targeted delivery of estrogen for the treatment of postmenopausal osteoporosis
Source: Mater Today Bio. 2023 Jul 26;22:100747. doi: 10.1016/j.mtbio.2023.100747 (PMC10415756; doi:10.1016/j.mtbio.2023.100747)
Supplement: Multimedia component 1 [file mmc1.docx]

Supplementray material

**Natural citric acid-modified pH-sensitive polymeric micelles loading 17β-Estradiol for the bone-targeting therapy**

**Table S1.** Encapsulation efficiency and loading efficiency of E2 in CA-PEOz@E2 at various weight ratios of E2/Polymer. 2:10 was chosen in this study.

| E2: Polymer | 1: 15 | 1: 10 | 2: 10 | 3: 10 | 4: 10 |
| --- | --- | --- | --- | --- | --- |
| EE | 93.34% | 84.54% | 77.72% | 61.41% | 48.79% |
| DLE | 5.91% | 7.79% | 13.19% | 13.60% | 14.37% |

**Table S2.** Characterization of different drug formulations by DLS.

|  | PEOz | PEOz@E2 | CA-PEOz | CA-PEOz@E2 |
| --- | --- | --- | --- | --- |
| Size | 103.4 ±7.8 | 109.2 ±9.4 | 112.6 ±5.7 | 119.5 ±11.2 |
| PDI | 0.214 ±0.04 | 0.202 ±0.08 | 0.158 ±0.07 | 0.217 ±0.07 |
| Zeta | -19.4 ±2.9 | -22.0 ±2.50 | -25.4 ±4.1 | -27.4 ±3.1 |


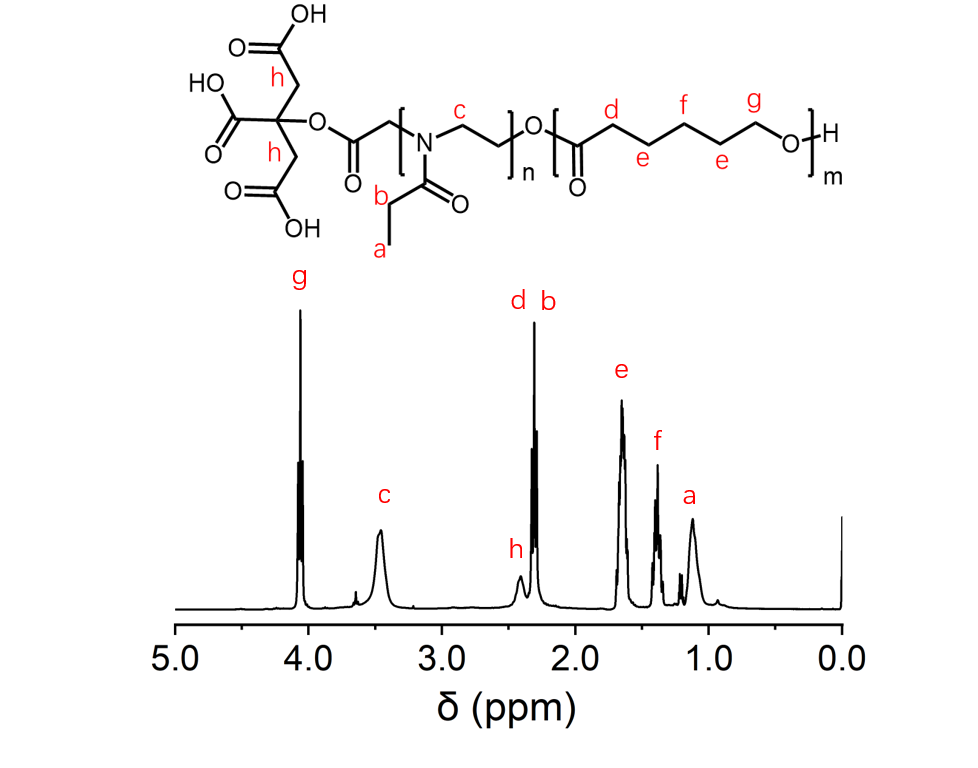


**Fig. S1** 1H NMR spectrum of CA-PEOz-PCL.





**Fig. S2** UV-Vis standard concentration curve of β-estradiol (E2) measured at 280 nm.
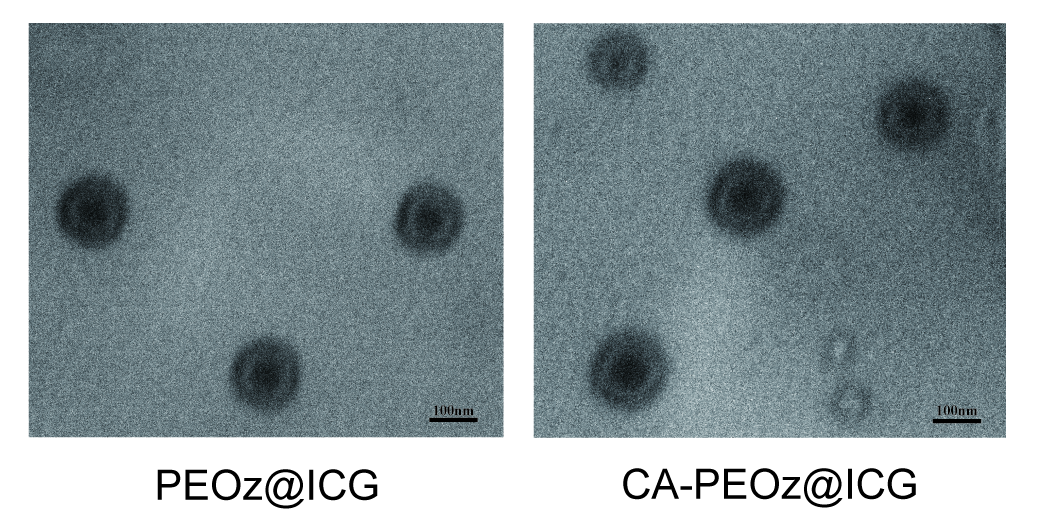


**Fig. S3** Morphological characteristics of PEOz@ICG and CA-PEOz@ICG by TEM. Scale bars represent 100μm. TEM shows that PEOz@ICG and CA-PEOz@ICG exhibit a stable spherical shape and uniform distribution.


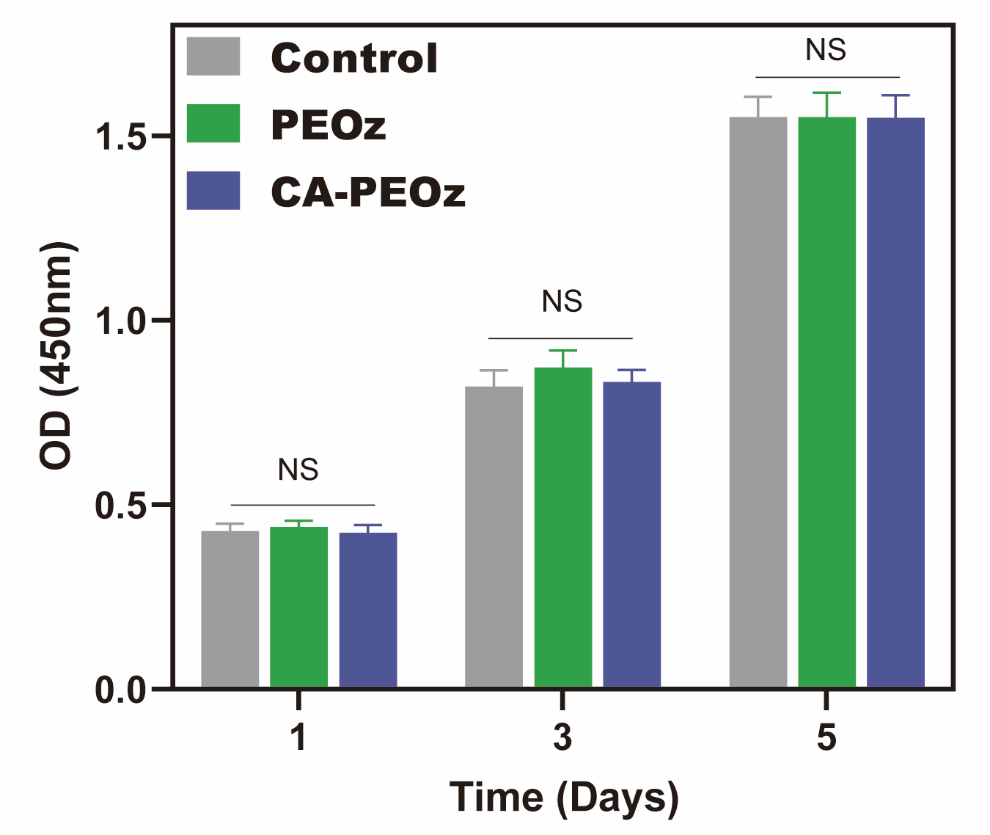


**Fig. S4** Cell viability of L929 cells cocultured with PEOz and CA-PEOz for 1, 3, and 5 days by CCK-8 assay (n = 3). NS mean no significant.


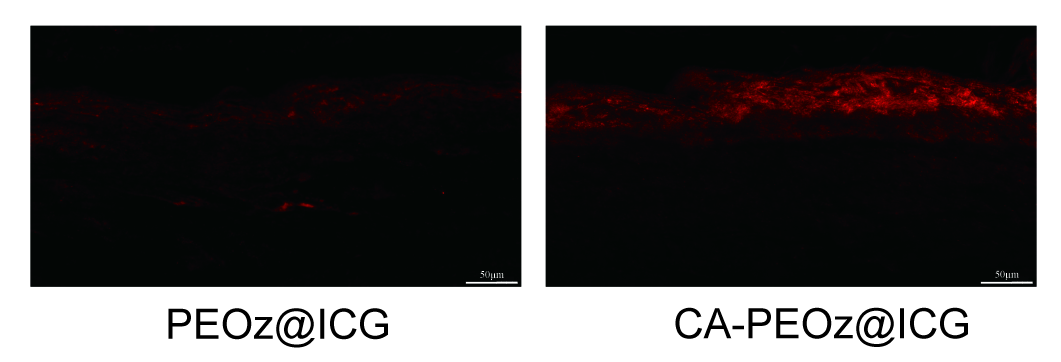


**Fig. S5** The histological distribution of the PEOz@ICG and CA-PEOz@ICG in bones. Scale bars represent 50μm.


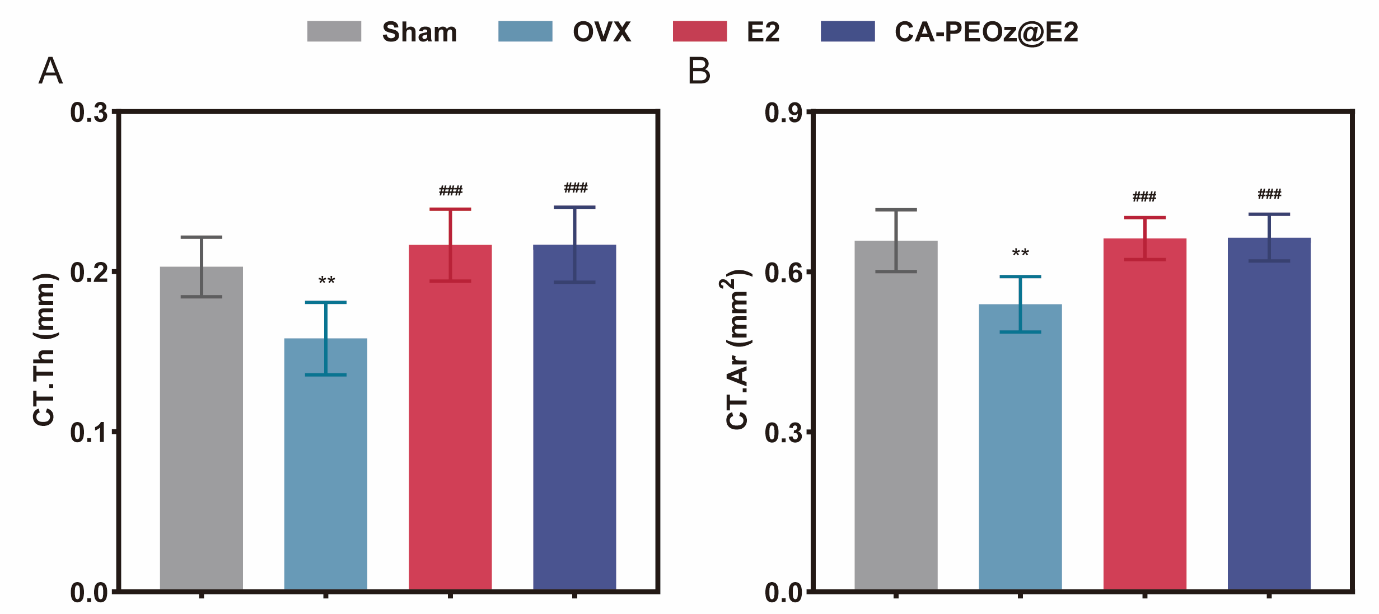


**Fig. S6** The quantification of femurs CT.Th and CT.Ar in each group. *: P <0.05, **: P < 0.01, and ***: P < 0.001. vs. the Sham. #: P <0.05, ##: P < 0.01, and ###: P < 0.001. vs. the OVX group.


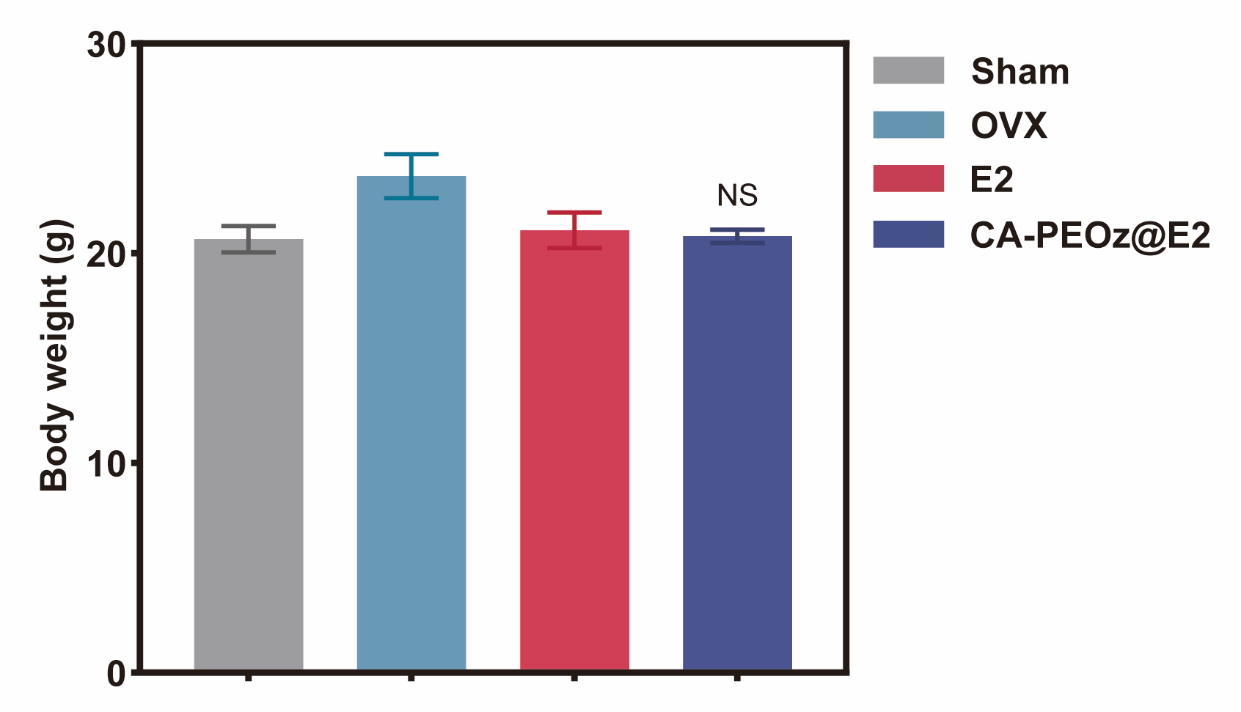


**Fig. S7** Body weight change of mice in each group at the end of the study. NS mean no significant.


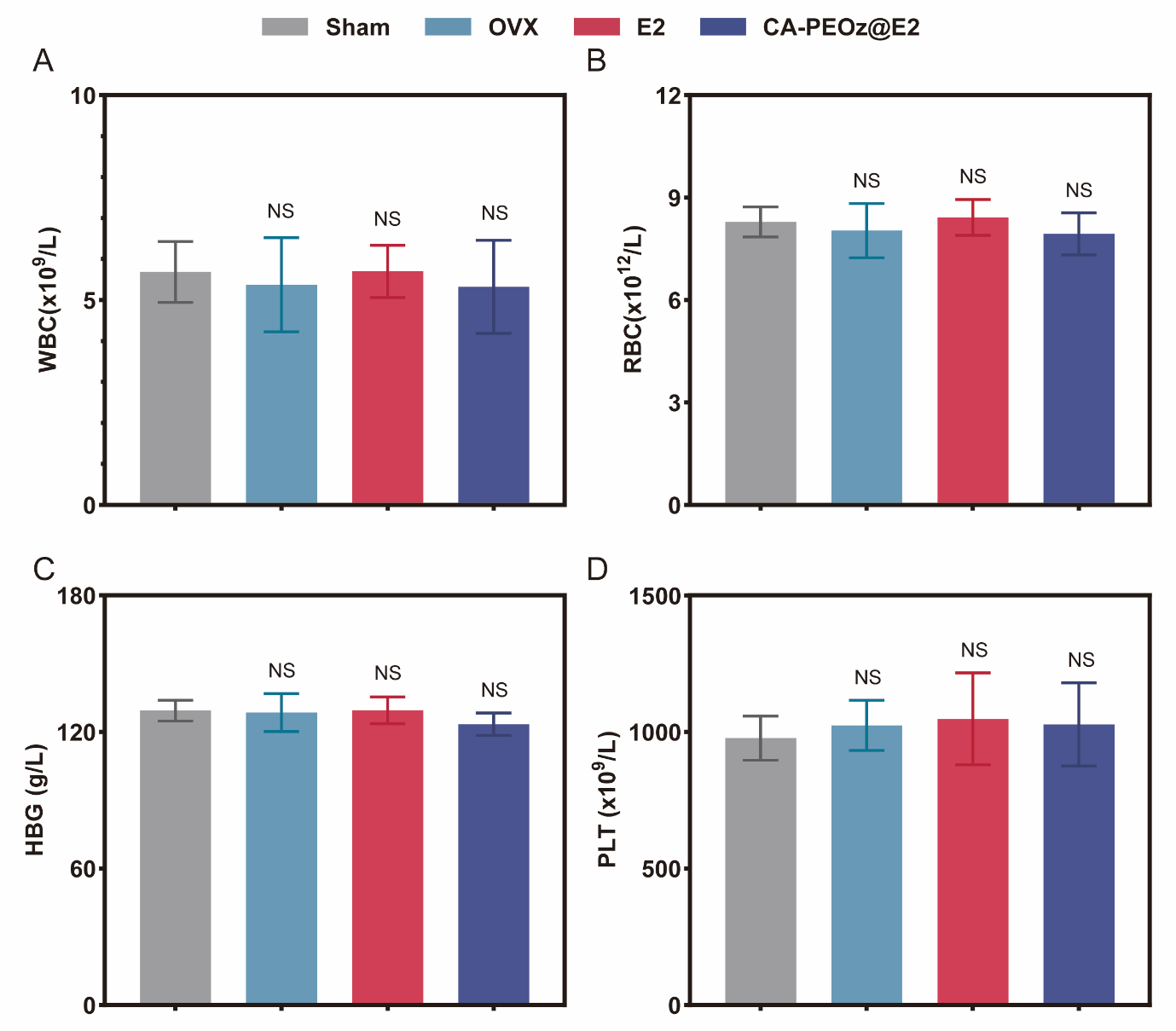


**Fig. S8** Effect of CA-PEOz@E2 on WBC, RBC, HGB, and PLT in each group at the end of the study. NS mean no significant.


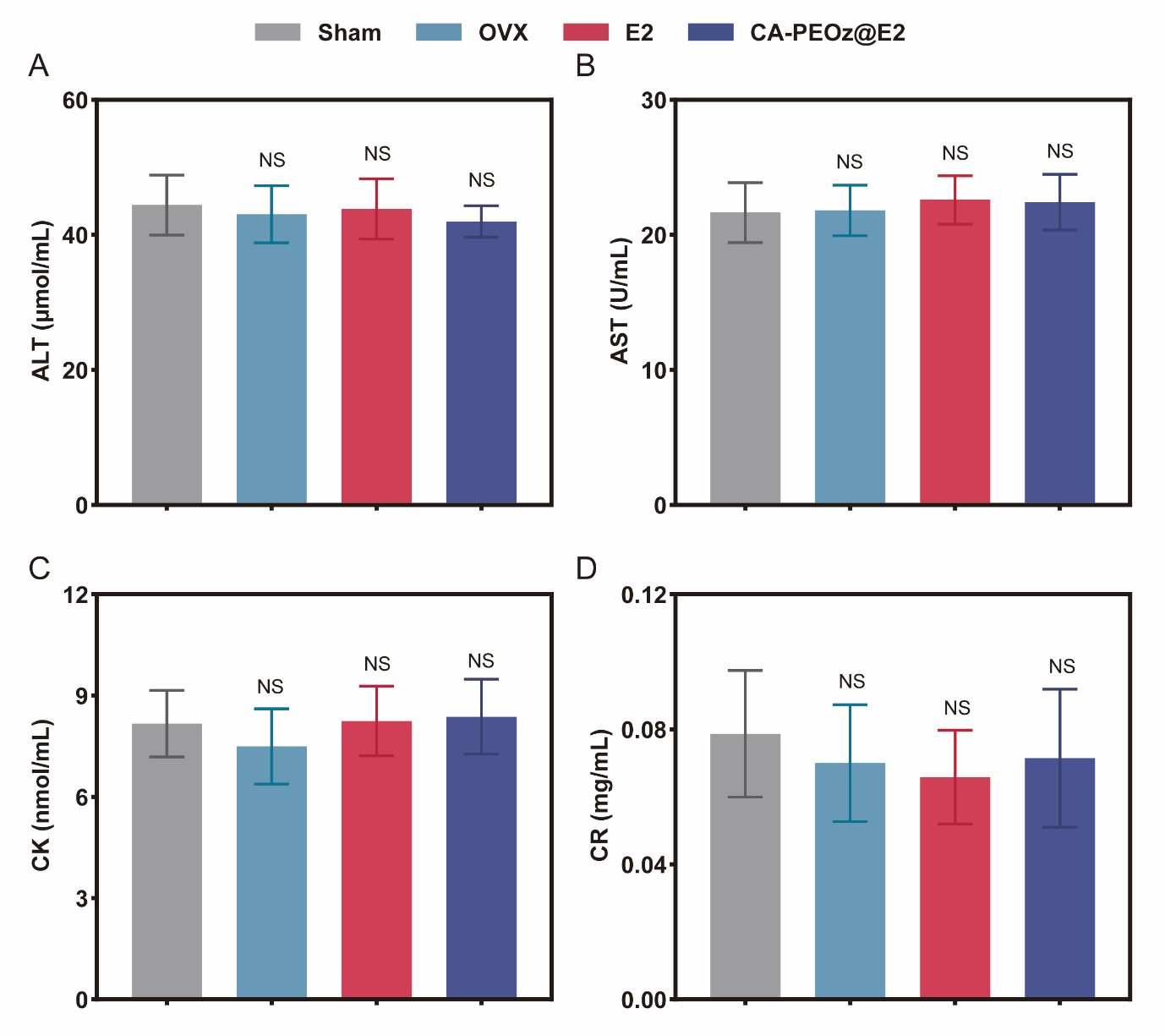


**Fig. S9** Impact of CA-PEOz@E2 on ALT, AST, CK, and CR in each group at the end of the study. NS mean no significant.


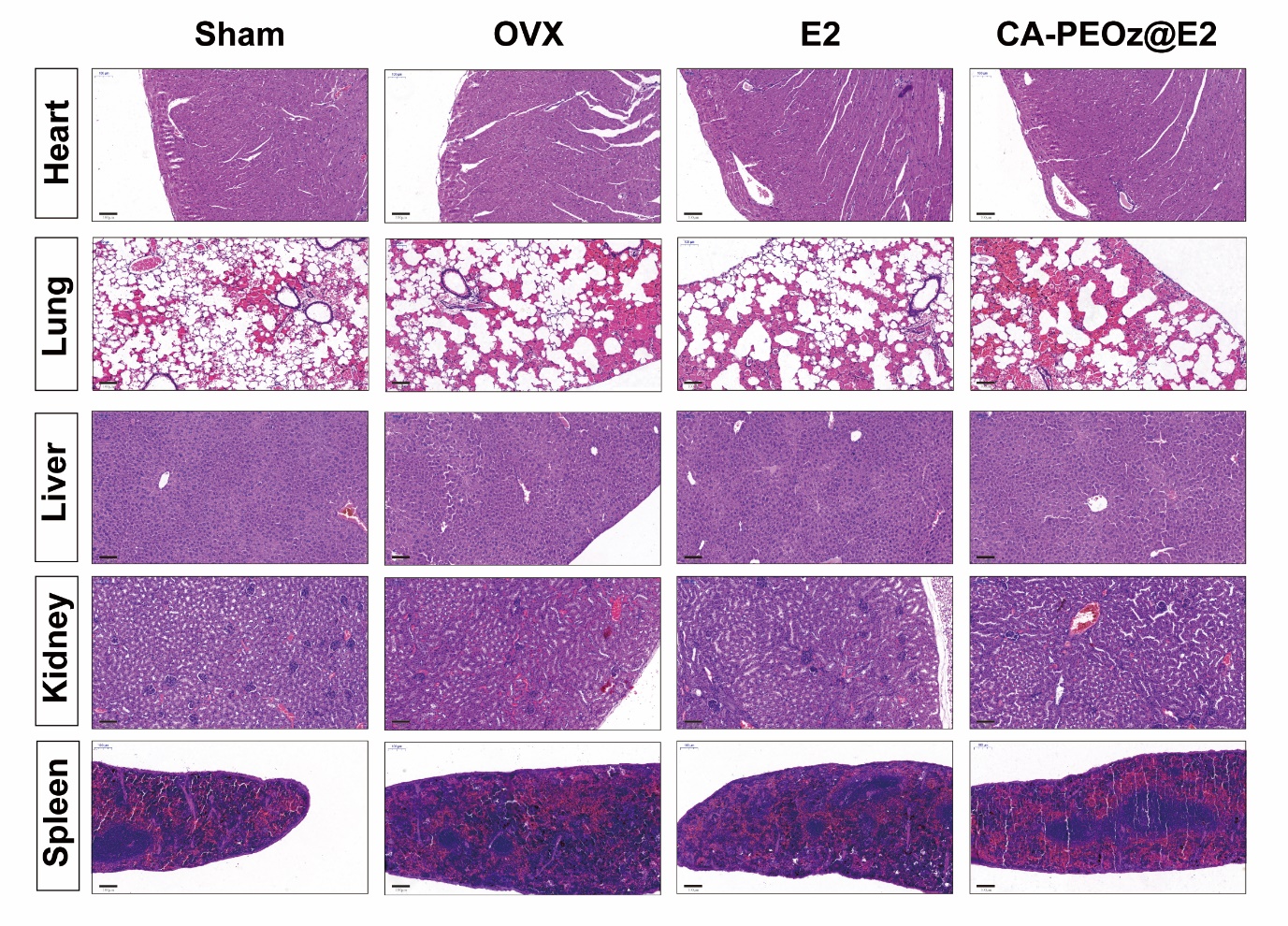


**Fig. S10** H&E staining evaluated systemic major organs (heart, liver, spleen, lung, and kidney) at the end of the study. Scale bars represent 100μm.
